# Pyriproxyfen and the microcephaly epidemic in Brazil - an ecological approach to explore the hypothesis of their association

**DOI:** 10.1590/0074-02760160291

**Published:** 2016-10-31

**Authors:** Maria de Fatima P Militão de Albuquerque, Wayner V de Souza, Antônio da Cruz G Mendes, Tereza M Lyra, Ricardo AA Ximenes, Thália VB Araújo, Cynthia Braga, Demócrito B Miranda-Filho, Celina MT Martelli, Laura C Rodrigues

**Affiliations:** 1 Fundação Oswaldo Cruz, Centro de Pesquisas Aggeu Magalhães, Departamento de Saúde Coletiva, Recife, PE, Brasil; 2 Universidade Federal de Pernambuco, Recife, PE, Brasil; 3 Universidade de Pernambuco, Recife, PE, Brasil; 4 London School of Hygiene and Tropical Medicine, London, UK

**Keywords:** Zika virus, microcephaly, pyriproxyfen larvicide

## Abstract

The microcephaly epidemic in Brazil generated intense debate regarding its causality, and one hypothesised cause of this epidemic, now recognised as congenital Zika virus syndrome, was the treatment of drinking water tanks with pyriproxyfen to control *Aedes aegypti* larvae. We present the results of a geographical analysis of the association between the prevalence of microcephaly confirmed by Fenton growth charts and the type of larvicide used in the municipalities that were home to the mothers of the affected newborns in the metropolitan region of Recife in Pernambuco, the state in Brazil where the epidemic was first detected. The overall prevalence of microcephaly was 82 per 10,000 live births in the three municipalities that used the larvicide Bti (*Bacillus thuringiensis israelensis*) instead of pyriproxyfen, and 69 per 10,000 live births in the eleven municipalities that used pyriproxyfen. The difference was not statistically significant. Our results show that the prevalence of microcephaly was not higher in the areas in which pyriproxyfen was used. In this ecological approach, there was no evidence of a correlation between the use of pyriproxyfen in the municipalities and the microcephaly epidemic.

One of the controversies regarding the aetiology of the microcephaly epidemic in Northeast Brazil, which was recognised to be associated with Zika virus infection (Rasmussen et al. 2016, WHO 2016), was the potential harm associated with the use of pyriproxyfen in drinking water tanks as a larvicide against *Aedes aegypti* (ABRASCO 2016). The Brazilian Ministry of Health (MoH) responded to the hypothesised association with the statement, “There are no epidemiological studies showing the association between use of pyriproxyfen and microcephaly”. Pyriproxyfen is a pyridine-based broad-spectrum larvicide that functions as an insect growth regulator and is effective against a variety of harmful insects. It was originally used in agricultural pest control (WHO 2008). Pyriproxyfen was approved as a pesticide by the World Health Organisation (WHO) and the Brazilian regulatory agency ANVISA. However, suspicions regarding its safety remained (Bar-Yam et al. 2016, Evans et al. 2016), and the state of Rio Grande do Sul, in Brazil, suspended the use of pyriproxyfen in February 2016 (EBC 2016). A recent review of the literature evaluating the safety of pyriproxyfen revealed a lack of data, preventing an adequate risk assessment of the potential role of pyriproxyfen in the induction of microcephaly (SWETOX 2016).

Pernambuco was the first state in Brazil to detect an increase in the number of cases of microcephaly compared to previous years, and it remains the region most affected by the epidemic (Brasil 2016). In Pernambuco, chemical larvicide (pyriproxyfen) or biological larvicide (*Bacillus thuringiensis israelensis* - Bti) were used in two distinct groups of municipalities, according to the vector control programs (CES 2016).

The current study aimed to explore the possible association between the prevalence of microcephaly, registered during the epidemic, and the use of chemical or biological larvicide in the metropolitan region of Recife (MRR), in Pernambuco state, Brazil.

The MRR is composed of 14 municipalities, with a population of 3,743,854 in 2012 (DATASUS - http://tabnet.datasus.gov.br/cgi/tabcgi.exe?ibge/cnv/poppe.def). According to the State Department of Health data from 2014 and 2015, three municipalities, Recife, Jaboatão and Paulista, used Bti, while the others employed pyriproxyfen (CES 2016). We obtained data regarding notifications of microcephaly from the State Department of Health Surveillance System for the period between 2 August 2015, and 3 April 2016. To define a case of microcephaly, we used Fenton growth curves with a cut-off of -2 standard deviations (SD) below the mean value for newborn head circumference (not the criteria bellow the 3rd percentile). This is very close, but not exactly the same as the intergrowth standards, which is now recommended to for the assessment of microcephaly (Victora 2016). The number of newborns in the study period was estimated as a proportion (eight months) of the annual number of births in the year 2013 by municipalities (SINASC)/MoH (DATASUS - http://tabnet.datasus.gov.br/cgi/deftohtm.exe?sinasc/cnv/nvpe.def). This number was used as the denominator to calculate the prevalence rates according to the mother’s municipality of residence. We tested the difference in the prevalence of microcephaly for the two groups of municipalities: those exposed to pyriproxyfen versus those not exposed.

We calculated the risk ratio and 95% confidence interval (CI) with a significance level given by the c^2^ test with Yates correction. During the study period, there were 719 reported cases, with 295 (41%) cases confirmed by the Fenton criteria.

Table presents, for each municipality, the proportion of newborns with microcephaly born to mother who were residents of that municipality and indicates whether pyriproxyfen was used in the drinking water tanks in that municipality. The three municipalities in which Bti was used had an overall prevalence of microcephaly of 82 per 10,000 live births, which was not statistically significantly different from the prevalence of 69 per 10,000 live births in the 11 municipalities that utilised pyriproxyfen. The risk ratio obtained was 0.85 (95% CI, 0.66-1.08; p = 0.21). Figure displays the spatial distribution of microcephaly prevalence per 10,000 live births according to the municipalities of the MRR.

**TABLE:** Prevalence of microcephaly according to the type of larvicide used by municipalities of Recife metropolitan region, Pernambuco, Brazil

Municipalities / larvicide	Cases	Newborns	Prevalence per 10,000
*Bacillus thuringiensis israelensis*			
Recife	124	15,451	80.3
Jaboatão	56	6,474	86.5
Paulista	22	2,756	79.8
Sub-total	202	24,681	81.8
Pyriproxyfen			
Olinda	18	4,013	44.9
Cabo de Sto. Agostinho	13	2,279	57.0
Camaragibe	14	1,572	89.1
S Lourenço da Mata	13	1,133	114.8
Igarassu	7	1,090	64.2
Ipojuca	14	1,066	131.3
Abreu e Lima	4	1,021	39.2
Moreno	5	543	92.0
Itapissuma	2	240	83.3
Araçoiaba	1	237	42.1
Ilha de Itamaracá	2	217	92.0
Sub-total	93	13,412	69.3

Total	295	38,093	77.4

x^2^ = 1.61; df = 1; p = 0.21

**Figure:**
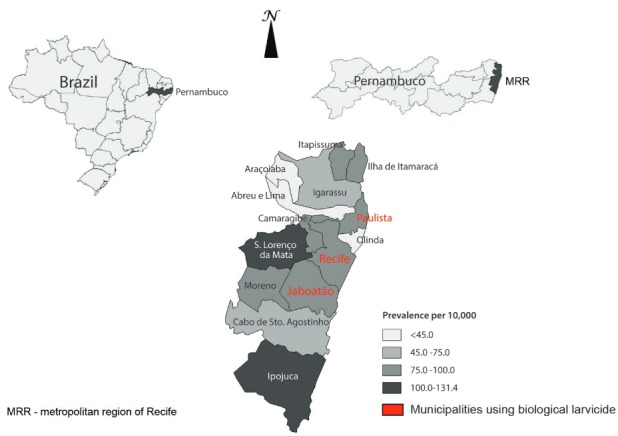
Prevalence of microcephaly per 10,000 live births according to municipalities of Recife metropolitan region, Pernambuco, Brazil.

Our results show that the prevalence of microcephaly was not higher in the areas in which pyriproxyfen was used. In this ecological approach, there was no evidence of a correlation between the use of this larvicide in the municipalities and the epidemic of microcephaly. This study is relevant to document the lack of an association, and we hope it is also a good example of the use of routine data to test a hypothesis. One limitation of this study is that the spatial units are large and populous. There may be a certain degree of heterogeneity in the vector control activities at the neighbourhood and house levels. Nevertheless, only one type of larvicide was used in each municipality during the reference period.

Considering that this was an ecological study, we do not have information regarding exposure on disaggregated levels. We compared areas exposed to pyriproxyfen to areas not exposed to pyriproxyfen to test if there was an association between pyriproxyfen and microcephaly at the ecological level. However, our findings do not invalidate the argument that improvements in environmental management to prevent and control disease may be a better choice than the widespread use of larvicide in drinking water for vector control, nor do they exclude other potential toxicities of pyriproxyfen (ABRASCO 2016). In addition, it should be noted that the mosquito vector control strategies during the past three decades, mainly based on chemical insecticides and larvicides, have proven ineffective (Regis et al. 2014, Augusto et al. 2016). Moreover, it is necessary to investigate other potential toxicities of pyriproxyfen. Despite approval from regulatory agencies (WHO 2006), further research may be needed to exclude other negative health outcomes, since during the Zika epidemic (and the potential emergence of other arboviruses), this product might be applied even more widely to drinking water for vector control. We emphasise that our findings seem timely because the analysis was performed in a region in which the microcephaly epidemic was most severe, and for which information was available on the use of chemical or biological larvicide by municipalities.
